# Dermatophytosis: Prevalence of Dermatophytes and Non-Dermatophyte Fungi from Patients Attending Arsho Advanced Medical Laboratory, Addis Ababa, Ethiopia

**DOI:** 10.1155/2018/8164757

**Published:** 2018-10-03

**Authors:** Adane Bitew

**Affiliations:** Department of Medical Laboratory Sciences, College of Health Sciences, Addis Ababa University, Addis Ababa, Ethiopia

## Abstract

**Background:**

Dermatophytosis is a disease of major public health problem around the globe causing a considerable morbidity.

**Objective:**

To study the prevalence of dermatophytosis and the spectrum of fungi implicated in causing the infection.

**Methods:**

Nail, skin, and scalp scrapings were collected from 318 patients and were used for microscopy and culture study. Fungal pathogens were identified by studying the macroscopic and microscopic characteristics of their colonies.

**Result:**

Tinea capitis was the predominant clinical manifestation consisting of 48.1% of the cases. Among 153 patients with tinea capitis, 73.2% were in the age group of 1-14 years. Of 318 study participants, 213 (67.98%) were found to be positive for dermatophytosis microbiologically. Out of 164 fungal isolates, 86 were dermatophytes and 78 were non-dermatophyte fungi. Among 86 dermatophytes,* T*.* violaceum* represented 38.4% of dermatophyte isolates and 89.7% of the isolates were recovered from tinea capitis. Of 76 non-dermatophyte molds,* Aspergillu*s spp.,* Scytalidium dimidiatum, and Cladosporium *spp. were the most common isolates, respectively.

**Conclusions:**

Failure to detect or isolate fungal pathogens in a large number of clinical samples revealed the limitation of clinical diagnosis in differentiating dermatophytosis from other skin infections demonstrating that clinical diagnosis should be coupled with laboratory methods. Recovery of large number of non-dermatophyte fungi along with dermatophytes in our study showed that non-dermatophyte fungi are emerging as important causes of dermatophytosis, warranting the implementation of intensive epidemiological studies of dermatophytosis across the country.

## 1. Introduction

Diseases caused by fungi can be divided into three broad groups: superficial mycosis, subcutaneous mycosis, and systemic mycosis. Among superficial mycosis, dermatophytosis is the most common contagious infection. It is a fungal infection of the outermost layer of skin and its appendages such as hair and nails with scalp ringworm being the most common in children of school age and adult males, respectively [1–4]. Dermatophytosis is currently a disease of worldwide importance and a public health problem in many parts of the world particularly in developing countries [5, 6]. Although the disease hardly causes death, it is a common refractory infection deleteriously affecting the quality of life via social stigma and upsetting day-to-day activities [1]. Large population size, low socioeconomic status, inadequate health facilities, and exchanging of foot-wears, clothes, and barbershop materials among people in developing nation have been recognized as potential risk factors for the proliferation of the disease [1, 4].

Although species of* Epidermophyton, Microsporum*, and* Trichophyton *are the major cause of the mycosis [5, 7], an infection of skin and its appendage by non-dermatophyte molds and yeasts has been increasing [8–12]. Emergence of chronic diseases such as diabetes that resulted from an increase in the life expectancy of world population and suppression of host immune defense mechanisms by underlying diseases have made humans more susceptible not only to pathogenic fungi but also to all fungi that were once considered contaminants [13, 14].

Dermatophytes and non-dermatophyte fungi implicated as a cause of dermatophytosis have been recorded all over the world, but with variation in distribution, incidence, epidemiology, clinical manifestations, and target hosts from one location to another. Differences in geographical location, health care, climatic factors, culture, and socioeconomic conditions are known to govern these discrepancies [15, 16].

In Ethiopia, studies conducted on dermatophytosis are few and these studies are concentrated on tinea capitis caused by dermatophytes primarily in children of school age [17–20]. There are only two studies of fungal infection of nails, skin, and scalp by dermatophytes and/or non-dermatophyte fungi [21, 22]. Furthermore, most of these studies were conducted before 2006. To this end, investigating human dermatophytosis regardless of age, site, and the distribution of fungi implicated in causing superficial mycosis appears to be one of the priorities in health related studies in Ethiopia.

## 2. Materials and Methods

### 2.1. Study Population

This prospective study was conducted from May 2017 to April 2018 at Arsho Advanced Medical Laboratory, Addis Ababa, Ethiopia. The study involved 318 patients that are clinically diagnosed for superficial mycosis and referred to Arsho from different health institutions for laboratory diagnosis.

### 2.2. Specimen Collection

Prior to sample collection, written informed consent was completed and signed by adult study subjects. Consent form was completed and signed by parents and/or guardians for those study subjects under 16 years of age. Patient information was collected using standard format. Nail, skin, and scalp scrapings were collected aseptically using sterile blades and transferred into sterile plastic petri-dishes.

### 2.3. Laboratory Diagnosis

Non-fungal elements were digested by placing clinical samples onto 20% potassium hydroxide (KOH) in a microscopic slide for about 5 to 10 minutes. The KOH preparation then was examined for the presence of fungal elements under low (×10) and high (×40) power magnification objective lenses. A portion of each clinical specimen was also streaked onto Mycosel agar and Sabouraud's dextrose agar containing chloramphenicol and gentamycin but without cycloheximide (BBL, Decton, Dickinsn and Company, USA). All plates were incubated at room temperature (25°C) for a minimum of 4 weeks supervising them frequently for any fungal growth. Fungi were then identified by studying the macroscopic and microscopic characteristics of their culture. Texture, rate of growth, topography, and pigmentation of the front and the reverse side of the cultures were employed to characterize fungi macroscopically. Lactophenol cotton blue mount of each fungal isolate was used to characterize fungal isolates microscopically. Occasionally, urease test was used in the differentiation of* T. tonsurans*,* T. violaceum*, and* T. rubrum.* Many mycological laboratory texts and manuals [23–25] were used as reference materials in process of identification. Yeasts were identified by means of conventional routine diagnostic methods [25] and chromogenic medium, CHROMagar Candida (bioMérieux, France) as per the instruction of the manufacturer.

*Ethical Clearance. *All ethical considerations and obligations were duly addressed. The study was carried out after the approval of the research and ethical committee of Arsho Advanced Medical Laboratory private limited company (AAMLRERC). Data collection was started after obtaining written informed consent from study subjects and assent form was completed and signed by parents and/or guardians. All the information obtained from the study subjects was coded to maintain confidentially.

## 3. Results

In this study, a total of 318 clinical samples were collected from suspected cases of dermatophytosis of which 122(38.4%) were from male and 196 (61.6%) from female patients. Tinea capitis was the predominant clinical manifestation consisting of 48.1% (153/318) of the cases. This was followed by tinea unguium and tinea corporis representing 18.9% (60/318) and 17.9% (57/318) of the cases, respectively (Table 1).

As shown in Table 2, out of 318 study subjects enrolled, fungi were detected and/or isolated in 213 (67.98%). One hundred thirty-one (41.2%) clinical samples were KOH positive while 154 (48.4%) clinical samples were culture positive. Mixed infections were observed in 3.1% (n = 10) of the culture positive cases. Fungi were neither detected nor showed visible fungal growth in 105 (33.3%).

As depicted in Table 3, clinical manifestation in relation to age was the highest in study subjects with age group of 1-14 (150) followed by age groups of 25-44 (98) and age groups of 15-24 (39), respectively. Out of 153 study subjects with tinea capitis, 73.2% (112/153) were ≤ 14 years of age. Study subjects in age group of 25-44 were the second most affected, tinea corporis being the highest. Tinea pedis was recorded in study subjects with an age of ≥ 25 years.

Among a total of 164 fungal isolates, dermatophytes were the most common isolates comprising 86 (52.4%) of the total isolates.* T. violaceum *was the dominant species involving 33 (38.4%) of the total dermatophyte isolates in which 29 (87.9%) of them were isolated from the scalp (Table 4). Seventy-eight isolates (47.6%) were non-dermatophyte fungi of which 69 (42.1%) were non-dermatophyte molds and the remaining 9 (5.5%) were yeasts.* Aspergillus *species (21),* Scytalidium dimidiatum (13),* and* Cladosporium *spp. (13) were the 1st and the 2nd common isolates of non-dermatophyte fungi (Table 5).* Scytalidium dimidiatum *wa*s *isolated from patients only with tinea corporis and tinea unguium.

## 4. Discussion

In the current study, the prevalence of dermatophytosis was high (66.98%). This is understandable, given that Ethiopia is a tropical country with wet humid climate, large population size, low socioeconomic status, and inadequate health facilities that are conducive for the proliferation of dermatophytosis. Strong correlations between dermatological infections and low socioeconomic conditions, geographical locations, climate, overcrowding, health care, and hygiene have been demonstrated by many researchers [26–28].

Fungi were not detected and isolated in 33.3% study subjects suspected of having superficial mycosis indicating that differentiation of dermatophytosis from other related superficial infections by clinical means only is not reliable. Coupling of clinical diagnosis with laboratory diagnosis appeared to be essential for better diagnosis as the cost and long duration of fungal therapy underline the significance of accurate diagnosis of the condition before starting therapy.

In our study, about seven different types of tinea were noted among which tinea capitis was the dominant clinical manifestation accounting for 48.1% of the total study subjects. According to Evans and Gentles [1], dermatophytosis affects both sexes, all ages, and all races, scalp ringworm being the predominant disease of children and tinea pedis being the predominant disease of adults, particularly adult males. Our result attested the work of Evans and his coworker [1] because, among study subjects with age ranging from 2 to 87 years, study subjects in the age range of 1-14 were the most affected with tinea capitis. Among 153 patients with tinea capits, 73.2% were in the age group of 1-14 years and tinea pedis was recorded in study subjects of ≥25 years of age. Differences in the amount of hormones before and after puberty [29] and insufficient production of fatty acids that have antifungal effect before puberty [30] are accountable for a difference in the prevalence of tinea capitis with age. Tinea capitis has been reported as the most frequent scalp infection affecting primary school children by previous studies conducted in Ethiopia [17–20]. These studies documented prevalence rates of tinea capitis in the range of 24.6-90%. In our study, tinea corporis, tinea unguium, and tinea pedis were less prevalent than tinea capitis. It has been reported that developing countries have high rates of tinea capitis, while developed ones have high rates of tinea pedis and onychomycosis [31]. High prevalence rates of tinea pedis and onychomycosis in developed countries have been related to increased urbanization of community showers, sports, and the use of occlusive footwear [16, 31, 32].

Out of 86 dermatophyte isolates in the present study, 69.8% were represented by* T*.* violaceum*,* T*.* mentagrophytes*, and* M. audouinii*,* T*.* violaceum* consisting of 38.4% of the total isolates and 89.7% isolated from patients with tinea capitis. Our finding was comparable with studies conducted in Ethiopia [17–20], many other African countries [33–35], and many Asian countries [36, 37]. According to Ameen [32],* T*.* violaceum *is an endemic dermatophyte in East Africa and Asia. Furthermore, 95.3% of the dermatophytes in our study were anthropophilic in contrast to developed countries where the major dermatophytes are zoophilic [38]. Differences in the mode of transmission of dermatophytes in developing and developed countries may explain the variation. In developing countries, transmission of dermatophytes from man to man is indirect via fomites (materials which are likely to carry infection, such as clothes, utensils, barbershop materials, and furniture). In addition to this, overcrowded human setting in developing countries has been noted as the main risk factor [37], whereas rearing and close proximity to domestic pets have been reported as significant risk factors for the transmission of dermatophytes in developed countries [39].

Non-dermatophytic molds were isolated from 44.8% culture positive study subjects, nails and skins being the most affected regions of the body. Our result was in line with the findings of Greer [40]. According to Greer [40], out of 691 nail infections, non-dermatophyte molds were recovered from 53% of the cases. The significance of non-dermatophyte mold species in skin-related infections has been highlighted in other many published studies [41–45]. However, the extent to which non-dermatophyte molds actually cause dermatophytosis particularly when a dermatophyte is present concurrently is still a subject of debate. Therefore, further investigations demonstrating how this group of fungi causes infection are needed.

Among non-dermatophyte molds isolated in the present study*, Aspergillus* species stood first. Our result supported the findings of Aikaterini et al. [46] and Nouripour-Sisakht et al. [47].

In the current study*, Scytalidium dimidiatum* represents a significant percentage of the non-dermatophyte mold isolates. They were isolated from skin and nail scrapings predominantly of toenails.* Scytalidium dimidiatum* and* Scytalidium hyalinum* are responsible for nearly 40% human superficial infections in tropical and subtropical regions [48].* Cladosporium *spp.*, Alternaria *spp.*, Fusarium *spp., and* Scopulariopsis brevicaulis* were other most commonly isolated non-dermatophyte molds recorded in our study. The significance of such non-dermatophyte molds in causing skin-related infections has been demonstrated in many other studies [49–51]. Similarly, C*andida albicans* has been isolated in 9 subjects with nail infection.* Candida albicans* as a major cause of tinea unguium has been documented in many studies [8–10, 42, 44, 46, 47].

## 5. Conclusions

Failure to detect or isolate fungal pathogens in a large number of clinical samples revealed the limitation of clinical diagnosis in differentiating dermatophytosis from skin infection caused by other organisms noting that clinical diagnosis should be coupled with laboratory methods. Recovery of large number of non-dermatophyte fungi along with dermatophytes in our study showed that non-dermatophyte fungi are emerging as important causes of dermatophytosis, warranting the implementation of intensive epidemiological studies of dermatophytosis across the country.

## Figures and Tables

**Table 1:** Frequency of clinical manifestation in relation to gender.

Clinical manifestation	Total number of samples	Sex	
	n (%)	Male n (%)	Female n (%)
Tinea capitis	153 (48.1)	63 (19.8)	90 ( 28.3)
Tinea corporis	57 (17.9)	19 (6.0)	38 (11.9)
Tinea unguium	60 (18.9)	21 (6.6)	39 (12.3)
Tinea pedis	14 (4.4)	4 (1.3)	10 (3.1)
Tinea faci	14 (4.4)	5 (1.6)	9 (2.8)
Tinea groin	8 (2.5)	6 (1.9)	2(0.6)
Tinea manum	12 (3.8)	3 (0.9)	9(2.8)

Total	318 (100)	122(38.4)	196(61.6)

**Table 2:** Correlation of direct microscopy and culture (n= 318).

Test procedure	Number	Percentage
KOH positive	131	41.2
Culture positive	154	48.4
KOH negative culture positive	75	23.6
KOH positive culture negative	55	17.3
KOH and culture positive	62	19.5
KOH and culture negative	105	33.3

**Table 3:** Frequency of clinical manifestation in different age groups (n=318).

Clinical manifestation	Total sample	Age groups				
		1-14	15-24	25-44	45-64	>65
Tinea capitis	153	112	9	24	7	1
Tinea corporis	57	15	8	28	4	2
Tinea unguium	60	13	18	22	6	1
Tinea pedis	14	-	-	9	4	1
Tinea faciei	14	7	1	5	1	-
Tinea manum	12	3	1	7	1	-
Tinea groin	8	-	2	3	2	1
Total	318	150	39	98	25	6

**Table 4:** Frequency and distribution of dermatophytes in relation to clinical manifestation.

Clinical presentation								
Fungal isolates	Tinea capitis	Tinea corporis	Tinea unguium	Tinea pedis	Tinea faciei	Tinea groin	Tinea manuum	Total
*T. violaceum*	29	2	2	-	-	-	-	33
*T. mentagrophytes*	5	2	3	4	-	-	1	15
*T. rubrum*	2	3	-	2	1	1	1	10
*T. tonsurans*	-	3	1	1	-	-	-	5
*T. soudanense*	1	1	-	1	-	1	-	4
*T. verrucosum*	2	-	-	-	-	-	-	2
*T. schoenleinii*	5	-	-	-	-	-	-	5
*M. audouinii*	8	2	-	-	1	-	1	12

Total	47	10	5	7	2	2	3	86

**Table 5:** Frequency and distribution of non-dermatophyte fungi in relation to clinical manifestations.

Clinical presentation								
Fungal isolates	Tinea capitis	Tinea corporis	Tinea unguium	Tinea pedis	Tinea faciei	Tinea groin	Tinea manum	Total
*Scytalidium dimidiatum*	-	2	10	-	1	-	-	13
*Cladosporium spp*	5	5	2	-	-	-	1	13
*Alternaria spp*	5	2	-	1	-	-	-	8
*Fusarium spp*	1	2	1	1	1	1	1	8
*Scopulariopsis brevicaulis*	-	1	4	-	-	-	1	6
*Phialophora*	-	3	-	-	-	-	-	3
*Exophiala*	1	2	-	-	-	-	-	3
*Exophiala werneckii*	-	-	-	-	-	-	2	2
*Fonsecaea spp*	-	1	-	-	-	-	-	1
*Aspergillus niger*	-	2	-	-	1	-	-	3
*Aspergellus fumigatus*	-	3	2	-	1	-	-	6
*A.teresus*	-	2	-	-	1	-	-	3
*Candida albicans*	1	1	7	-	-	-	-	9

Total	13	26	26	2	5	1	5	78

## Data Availability

The data used to support the findings of this work are available from the author upon request.
